# TcMYC2a, a Basic Helix–Loop–Helix Transcription Factor, Transduces JA-Signals and Regulates Taxol Biosynthesis in *Taxus chinensis*

**DOI:** 10.3389/fpls.2018.00863

**Published:** 2018-06-21

**Authors:** Meng Zhang, Xiaofei Jin, Ying Chen, Mi Wei, Weifang Liao, Shengying Zhao, Chunhua Fu, Longjiang Yu

**Affiliations:** ^1^Department of Biotechnology, Institute of Resource Biology and Biotechnology, College of Life Science and Technology, Huazhong University of Science and Technology, Wuhan, China; ^2^Key Laboratory of Molecular Biophysics, Ministry of Education, College of Life Science and Technology, Huazhong University of Science and Technology, Wuhan, China

**Keywords:** TcMYC2a, TcJAZ3, JA signaling pathway, *tasy* gene, *Taxus chinensis*

## Abstract

The multitherapeutic taxol, which can be obtained from *Taxus* spp., is the most widely used anticancer drug. Taxol biosynthesis is significantly regulated by jasmonate acid (JA), one of the most important endogenous hormones in land plants. Nevertheless, the JA-inducing mechanism remains poorly understood. MYC2 is one of the key regulators of JA signal transfer and the biosynthesis of various secondary metabolites. Here, TcMYC2a was identified to contain a basic helix–loop–helix (bHLH)-leucine zipper domain, a bHLH-MYC_N domain, and a BIF/ACT-like domain. TcMYC2a was also found to bind with TcJAZ3 in yeast, which was a homolog of *Arabidopsis* JASMONATE ZIM-domain JAZ proteins, indicating that TcMYC2a had a similar function to AtMYC2 of JA signal transduction. TcMYC2a was able to affect the expression of GUS reporter gene by binding with the T/G-box, G-box, and E-box, which were the key *cis*-elements of *TASY* and *TcERF12/15* promoter. *TcMYC2a* overexpression also led to significantly increased expression of *TASY*, *tat*, *dbtnbt*, *t13h*, and *t5h* genes. Additionally, TcERF15, which played the positive role to regulate *tasy* gene, was up-regulated by TcMYC2a. All these results revealed that TcMYC2a can regulate taxol biosynthesis either directly or via ERF regulators depending on JA signaling transduction.

## Introduction

**FIGURE 1:**
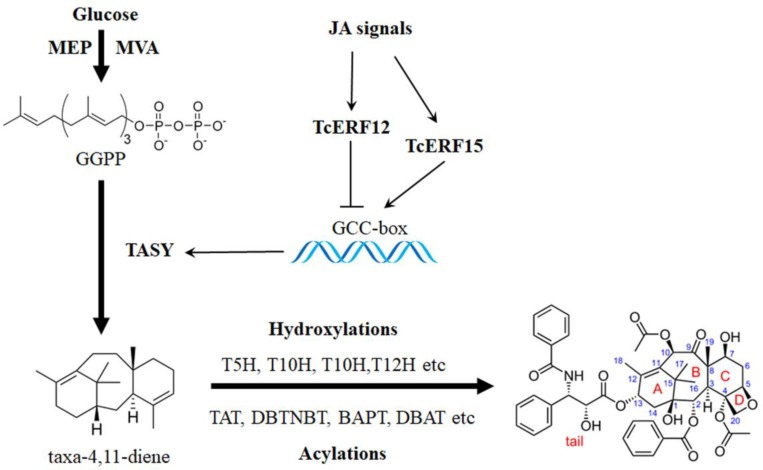
Transcription regulation of taxol biosynthesis. Current progress of transcription regulation of taxol biosynthesis. TASY (taxadiene synthase), T5H (taxadiene 5-alpha hydroxylase), TAT (taxadienol acetyltransferase), T10H (5-alpha-taxadienol-10-beta-hydroxylase), T13H (13-alpha-hydroxylase gene), TBT (taxane 2-alpha-*O*-benzoyltransferase), DBTNBT (3′-*N*-debenzoyltaxol *N*-benzoyltransferase), BAPT (phenylpropanoyltransferase), DBAT (10-deacetylbaccatin III-10-*O*-acetyltransferase) and PAM (phenylalanine aminomutase).

Taxol is widely used as an essential anticancer medicine for the effective clinical treatment of ovarian cancer, breast cancer, lung cancer, Kaposi sarcoma, cervical cancer, and pancreatic cancer, among others (Schiff et al., 1979). More than 20 enzymes are involved in catalyzing geranylgeranyl pyrophosphate to taxol, and the biosynthesis pathway is highly complex (**Figure 1**; Croteau et al., 2006). Nearly all known taxol-biosynthesis genes are up-regulated by JA and its derivatives, such as *TASY*, *DBAT*, *DBTNBT*, *T7H*, *T2H*, etc., but its mechanism is poorly understood (**Figure 1**) (Zhang et al., 2015b; Du et al., 2017).

Jasmonate acid (JA) is a vital endogenous hormone that regulates taxol biosynthesis and other biological processes, especially (a)biotic stress tolerance in various plants (Roberts, 2001). The physically interactions of JASMONATE ZIM-domain (JAZ) with several downstream transcription factors reportedly control the JA signal-transduction system, also called the JA signaling pathway (Deshaies, 1999; Thines et al., 2007; De Geyter et al., 2012). These downstream transcription factors are functionally disabled by physical interaction with JAZ until JA signaling molecules release them (Chini et al., 2016; Zhang et al., 2017). Among all JA downstream transcription factors, MYC2 is considered as the fatal point of the entire JA signaling pathway (Kazan and Manners, 2013).

MYC2, which belongs to clade IIIe of the basic helix–loop–helix (bHLH) superfamily that is one of the largest group of transcription factors in plants, is the master JA regulator involved in secondary metabolite biosynthesis (Heim et al., 2003; Chini et al., 2007, 2016; Schweizer et al., 2013; Berens et al., 2017). MYC2 is essential for JA response to secondary-metabolite accumulation in several plants. In *Catharanthus roseus*, CrMYC2 activates the expression of *ORCA3* gene, which responds to JA signal and functions as a crucial activator of *STR* gene, resulting in vinblastine accumulation (van der Fits and Memelink, 2000; Zhang et al., 2011). In *Nicotiana tabacum*, NtMYC2a/b, which can bind with NtJAZ1/2/3, is able to activate the expression of the following key genes of nicotine biosynthesis: *putrescine N-methyltransferase* (*PMT*), *quinolinate phosphoribosyltransferase* (*QPT2*), and *NtA622*. NtMYC2a/b can also bind ERF189 and ERF221, which are the major JA-responsive activators of *PMT2* (De Boer et al., 2011; Shoji and Hashimoto, 2011; Zhang et al., 2012). In *Artemisia annua*, AaMYC2 binds with AaJAZ1-4 and activates *CYP71AV1* and *DBR2* genes, which encode enzymes of artemisinin biosynthesis (Shen et al., 2016). In *Salvia miltiorrhiza*, SmMYC2a/b and SmJAZ1/2 can form a dimer complex, and the contents and expression of genes of tanshinones and phenolic acids are repressed in SmMYC2a/b knockdown plant lines. Thus, SmMYC2a and SmMYC2b are positive regulators (Zhou et al., 2016). In rice, JA-inducible OsMYC2 drastically enhances the activity of naringenin 7-O-methyltransferase (*OsNOMT*) promoter and is essential for JA-inducible sakuranetin production (Ogawa et al., 2017b). All these studies indicate that MYC2 is a vital regulator of secondary-metabolite biosynthesis and that the JAZ–MYC2 complex is critical to the JA signaling pathway.

*Taxadiene synthase* (*TASY*) promoter has been obtained and characterized, and the fragment containing the GCC-box at -142 bp is found to be the minimum region responding to JA signals. Thus, TcERF12 and TcERF15 are JA-responsive ERF factors that negatively and positively regulate *TASY* gene expression by binding with the GCC-box (Zhang et al., 2015b). However, how TcERF12 and TcERF15 respond to JA signals is unclear. Our previous study has shown that *TASY* promoter has a T/G- (AACGTG; -256 bp) and a G-box (CACGTG, -158 bp), both of which are MYC2 binding *cis*-elements. The two *cis*-elements contribute to JA responses, indicating that MYC2 is a candidate regulator of taxol biosynthesis responding to JA (Zhang et al., 2015b).

In the present work, a MYC2 transcription factor was screened in *Taxus chinensis* and named as TcMYC2a. Homolog search and phylogenic analysis identified it is a typical MYC2 transcription factor, and conserved domains and motifs showed its potential functions in JA signaling pathway. Yeast two-hybrid experiments verified the interaction of TcMYC2a with *Taxus* JAZ protein. Then, a series of experiments would be performed to determine the function of TcMYC2a in taxol biosynthesis and explain the regulation system.

## Materials and Methods

### RNA Isolation and cDNA Synthesis

*Taxus chinensis* cell line #48 was established using the callus cultures derived from a budding *Taxus* stem in May 2003 and was maintained in modified Gamborg’s B5 medium (Zhang et al., 2000). RNA isolation were conducted by RNAprep Pure Plant Kit (TIANGEN, Beijing, Cat: DP441), and the first chain of cDNA were transcribed by ReverTra Ace^®^ qPCR RT Master Mix with gDNA Remover kit (Toyobo, Japan, Code No. FSQ-301).

### Screening, Sequence Alignment and Phylogenetic Analyzing of TcMYC2a

The HMM files of HLH (Accession NO.: PF00010), bHLH-MYC_N (Accession NO.:PF14215) and ACT (Accession NO.: PF01842) domains were retrieved from the Pfam database^1^. Then, the HMMsearch program of HMMER package^2^ was used to identify the MYC2 proteins from our previous transcriptome datasets in *T. chinensis* (Zhang et al., 2015a,b; Liao et al., 2017). Specific primers (**Supplementary Table S1**) were designed to amplify *TcMYC2a* from the cDNA of *T. chinensis* under the following PCR parameters: initial denaturation at 95°C for 6 min; 30 cycles of denaturation at 94°C for 30 s; annealing at 58°C for 30 s; extension at 72°C for 2 min; and a final extension at 72°C for 10 min. The PCR products were subcloned into pMD18-T (TaKaRa, Japan) for sequencing. BLAST search^3^ was used for the homology search from Swiss-prot database. ClustalW online^4^ at the default setting was used to align and evaluate the percentage identity of TcMYC2a with known MYC2s. the alignment results were send to ESPript 3.0^5^ for coloring (Robert and Gouet, 2014). Phylogenetic analysis was performed with a neighbor-joining (NJ) method by using 1000 bootstrap resamplings in MEGA 5.05.

### Subcellular Localization

TcMYC2a was inserted into pCAMBIA1300-sGFP with *Kpn* I and *Sal* I restriction sites, then recombinant vector was transformed into LBA4404. 1 cm × 1 cm size onion epidermal cells were infected by positive clones in 1/2 MS liquid medium at 25°C, dark for 2 days. The infected onion epidermal cells were then plated on MS medium for 2 days at 25°C in darkness. The GFP fluorescence was observed by Laser scanning confocal microscope FV1000 (OLYMPUS, Japan).

### Yeast One-Hybrid and Yeast Two-Hybrid

TcMYC2a was amplified using primers as TcMYC2ayF and TcMYC2ayR (**Supplementary Table S1**), then purified PCR product and pGADT7-Rec2 plasmid were digested by *EcoR* I and *Xma* I enzymes at the same time. T4 ligase was used to link the two fragments to construct the prey vector, TcMYC2a-pGADT7R2. For constructing bait vector, sense and antisense sequences of quadrupled T/G-box, G-box and E-box were artificially synthesized, respectively (**Supplementary Table S1**). The cohesive ends of *Eco*R I and *Spe* I would expose after annealing, then the double strand were linked with digested pHIS2.1 vector to form pbaitT/G and pbaitG, respectively. Finally, TcMYC2a-pGADT7R2 were co-transformed with two bait vector, respectively, into the yeast strain Y187, and Y187 cells were plated onto SD/Leu-/Trp-/His-deficient medium with 40 mM 3-AT for 3–5 days.

TcMYC2a-pGADT7R2 and pGADT7 were digested by *EcoR* I and *Xma* I, the coding region of TcMYC2a were inserted into pGADT7 to construct TcMYC2a-pGADT7. TcJAZ3 (Zhang et al., 2017) was inserted into pGBKT7 with *Eco*R I and *Bam*H I restriction sites for TcJAZ3-pGBKT7. Then TcMYC2a-pGADT7 and TcJAZ3-pGBKT7 were co-transformed into AH109 using LiAc conversion protocol. The tranformants were screened on SD/Leu-/Trp-/His-deficient medium.

### Construction of Overexpression, RNAi Vector and Reporter Vectors

The coding region of TcMYC2a was inserted into the pCAMBIA1303 vector with *Nco* I and *Bst*E II restriction sites under control of the CaMV 35S promoter, resulting in the overexpression of *TcMYC2a* in *Taxus* cells. To construct TcMYC2a-RNAi vector, a 400 bp 3′ ORF sequence of TcMYC2a was inserted into pCAMBIA1300-35S-X forward (BamHI) and reverse (SpeI) so that SPLRNAi fragments were stuck between them. The primers were showed in **Supplementary Table S1**. The construct was transformed into the *T. chinensis* cells while the pCAMBIA1303 was used as the control. Transformation experiments were performed using three independent *T. chinensis* cell lines.

The mTtsp fragment was amplified used primers mTtspF and tspR with the *TASY* promoter as template. The mGtsp fragment was synthesized by Sangon Biotech (Shanghai) Co., Ltd., then the mGtsp and mTGtsp were amplified used primers mGtspF/mTGtspF and tspR with the mGtsp fragment as template. After then, mTtsp, mGtsp and mTGtsp replaced the 35S promoter of pBI121 with *Bam*H I and *Hind* III restriction sites, resulting a series of reporter vectors.

### Analysis of TcERF12 and TcERF15 Promoters

Firstly, the *T. baccata* genome sequences (Nystedt et al., 2013) were made a local database by the software^6^ BLAST+. Then, the ORF sequences of *TcERF12/15* were used as the reference gene to blast with the local database at the default parameters. Therefore, the homolog sequences containing 5′ upstream sequences and the ORF sequences were obtained. According to the homolog sequences, the specific forward primers were designed, and the specific reverse primer were designed according to ORF (**Supplementary Table S1**). Genome DNA was extracted by SDS-CTAB method and then used as a template. The specific product was T/A cloned, sequenced and aligned with designed partial sequence of TcERFs’ ORF to decide the 5′-flanking region. The 5′-flanking sequence was analyzed online by PlantCare^7^ and PLACE^8^ at default settings.

### Transient Transformation and GUS Activity Analysis

For overexpression of *TcMYC2a*, the TcMYC2a-p1303 vector was transformed into *T. chinensis* suspension cells mediated by Agrobacterium LBA4404, the detailed method referred to Zhang et al. (2015b). For co-overexpression, pTG, pG, pmTG, and pmG were co-transformed with TcMYC2a-p1303 into *Taxus* cells, respectively, quantitative analysis of GUS activity was conducted as our previous reports (Li et al., 2013). Each construction had three independent experiments. The transient transformations were conducted three times independently to make sure there were three independent biological repeats.

### Quantitative Real-Time PCR

1 μl synthesized cDNA (diluted 1:5) was used as template for real-time PCR. Beta-actin expression was used to normalize all values in the RT-PCR assays in *T. chinensis*. Primers for RT-PCR were designed using Primer5 software (**Supplementary Table S1**). SYBR Green Realtime PCR Master Mix (Toyobo, Japan) was used to conduct qRT-PCR, StepOnePlus (Applied Biosystems, United States) was used to detect and analyze the reactions. To enable statistical analysis, three fully independent biological replicates were obtained, each experiment was conducted three times, and we used Student’s *T*-test.

### HPLC (High Performance Liquid Chromatography)-MS (Mass Spectrum) Analysis of Taxanes

The transformation cells were 125 rpm shake at 25°C darkness for 6 days. Preparation of samples and HPLC analysis procedures were conducted as described previously (Song et al., 2014; Dong et al., 2015).

## Results

### TcMYC2a Shares Common Domains With MYC2 in Other Plants

**FIGURE 2:**
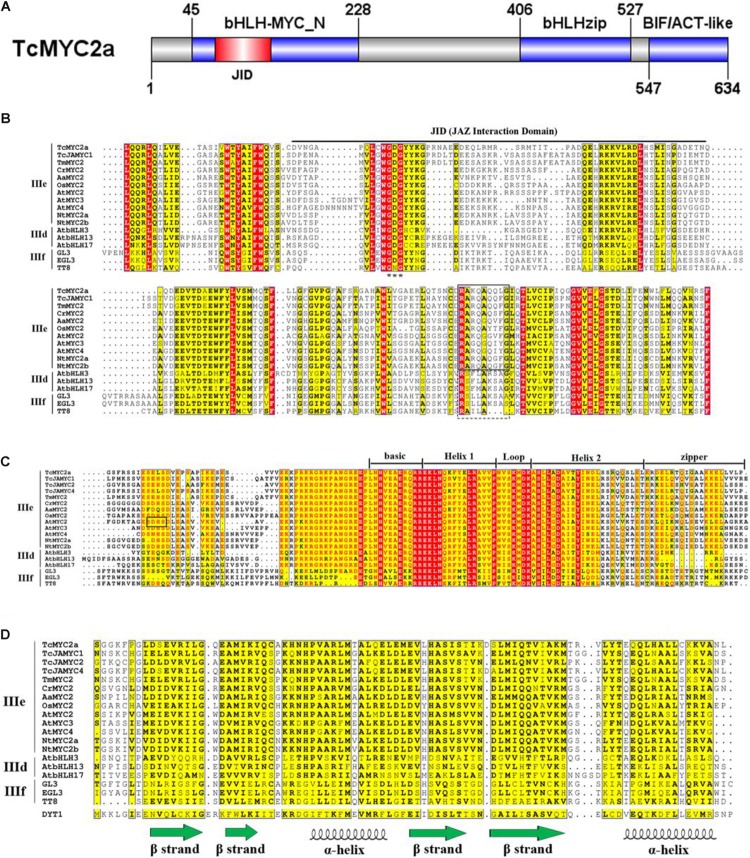
Molecular structure of TcMYC2a. Molecular structure of TcMYC2a and alignment of its conserved domains. **(A)** Sketch map of the molecular structure of TcMYC2a protein. bHLH-MYC_N, bHLHzip and BIF/ACT-like domain were blue boxes, and the JID (JAZ interaction domain) was in red. **(B)** Alignment of the bHLH-MYC_N domain of TcMYC2a with other clade IIId/IIIe/IIIf bHLHs. JID domain was underlined; GDG (Gly-Asp-Gly) residues were emphasized by stars; RA(K/R)QAQQ(F/Y)LG- and RSLLA**K**S**A**S-motif were by solid and dotted boxes, respectively. **(C)** Alignment of the bHLHzip domain. The basic region, helixes, loop and zip regions were marked. **(D)** Alignment of the BIF/ACT-like domain. The secondary structure (ββαββα) of this domain was visualized. Accession NO. in GenBank: TcMYC2a (MG494378), TcJAMYC1/2/4 (ACM48567.1/AGO03813.1/AGO03814.1), TmMYC2 (AHL44338.1), AtMYC2/3/4 (OAO98324.1/OAO98324.1/OAO98324.1), CrMYC2 (OAO98324.1), OsMYC2 (OAO98324.1), AaMYC2 (AKO62850.1), NtMYC2a/b (NP_001313001.1/NP_001312960.1), AtbHLH3/13/17 (AAL55710.1/AHL44338.1/Q9ZPY8.2), GL3 (NP_193864.1), EGL3 (AHL44338.1) TT8 (OAO98324.1) and DYT1 (OAO98340.1).

After homolog searching in the Swiss-Prot database, TcMYC2a was found to share the highest similarity with OsMYC2, which is a transcriptional activator involved in JA signaling pathway during spikelet development (Cai et al., 2014; Ogawa et al., 2017a). Sequence alignment showed that TcMYC2a contained all of the essential domains and motifs of MYC2 (**Figure 2**). Three conserved domains/motif were highly conserved in both TcMYC2a and other MYC2 factors: bHLH-zip-, bHLH-MYC_N-, and BIF/ACT-like-domain (Pauwels and Goossens, 2011).

Compared with GL3, EGL3, and TT8 (clade IIIf), TcMYC2a shared a more similar bHLH-zip domain and 14 aa co-adjacent N-terminal peptides with other MYC2s (clade IIIe) and clade IIId bHLHs. Clade IIId and IIIe are always considered as one subgroup because they function complementarily and redundantly (Feller et al., 2011; Tominaga-Wada et al., 2011). Additionally, nearly all bHLH-zip domains of MYC2 factors had a SELS peptide (414 aa–417 aa) similar to the SDHS peptide of AtMYC2, which was assumed to be the phosphorylation site. This 4aa-length peptide was only highly similar among MYC2s but highly variable in clades IIId and IIIf (**Figure 2D**) (Kazan and Manners, 2013).

Except for TcJAMYC2 and TcJAMYC4, the other clade IIId/e/f bHLHs including TcMYC2a contained the bHLH-MYC_N-domain (**Figure 2B**). Our results showed that TcMYC2a had a Gly-Asp-Gly motif, as well as other JAZ-interactive bHLHs (IIId-f), indicating that TcMYC2a can bind with JAZ proteins (Goossens et al., 2015). Additionally, non-MYC2 factors (IIId/f) contained a RSLLA**K**S**A**S-motif, in which K and A residues determined the transactivation strength of proteins, whereas all MYC2s had the RA(K/R)QAQQ(F/Y)LG-motif at the corresponding positions in the bHLH-MYC_N-domain. Meanwhile, TcMYC2a contained a RAKQAQQLG-motif that was highly homologous with MYC2 factors, indicating that TcMYC2a was a MYC2 homolog.

The aspartokinase, chorismate, and TyrA (ACT) domain was first found in a wide range of metabolic enzymes and formed the βαββαβ secondary structures (Chipman and Shaanan, 2001). The ACT domain of bHLH was predicted to have a ββαββα structure that differed from the βαββαβ structure (Chang et al., 2017). According to our results, the BIF/ACT-like domain was present in all subgroup IIId-IIIf bHLHs, and the similarity of BIF/ACT-like domain was low among the three clades (**Figure 2D**). TcMYC2a contained the BIF/ACT-like domain that was more highly similar to MYC2 proteins, indicating that TcMYC2a was more closely related to MYC2s.

All these results showed that TcMYC2a was preferred as a MYC2 transcription factor and that highly conserved domains enabled TcMYC2a to share similar functions to other MYC2s.

### Phylogenic Analysis Grouped TcMYC2a With Other MYC2 Proteins

**FIGURE 3:**
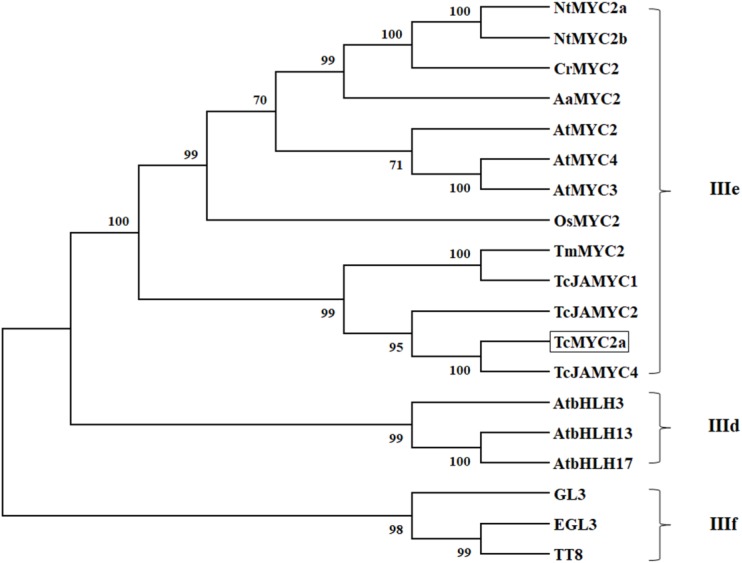
Phylogenetic tree of TcMYC2a. Phylogenetic analysis of full-length TcMYC2a and other clade IIId/IIIe/IIIf bHLHs; TcMYC2a was framed by a solid box. Accession NO. in GenBank: TcMYC2a (MG494378), TcJAMYC1/2/4 (ACM48567.1/AGO03813.1/AGO03814.1), TmMYC2 (AHL44338.1), AtMYC2/3/4 (OAO98324.1/OAO98324.1/OAO98324.1), CrMYC2 (OAO98324.1), OsMYC2 (OAO98324.1), AaMYC2 (AKO62850.1), NtMYC2a/b (NP_001313001.1/NP_001312960.1), AtbHLH3/13/17 (AAL55710.1/AHL44338.1/Q9ZPY8.2), GL3 (NP_193864.1), EGL3 (AHL44338.1) TT8 (OAO98324.1) and DYT1 (OAO98340.1).

Thirteen MYC2s, three clade IIIf (TT8, EGL3, and GL3), and three clade IIId bHLH were selected for phylogenic analysis. Twenty proteins were grouped into three clades, the MYC2 clade (IIIe), clade IIId, and clade IIIf (**Figure 3**). TT8, EGL3 and GL3, and AtbHLH3/13/17 were grouped together in accordance with previous reports (Tominaga-Wada et al., 2011). In the present work, TcMYC2a was grouped with MYC2 proteins and was most closely related to TcJAMYC4, which was identified from *T. cuspidate* although TcJAMYC4 lacked most of the entire bHLH_MYC-N domain (Lenka et al., 2015).

### TcMYC2a Localized in Nucleus

**FIGURE 4:**
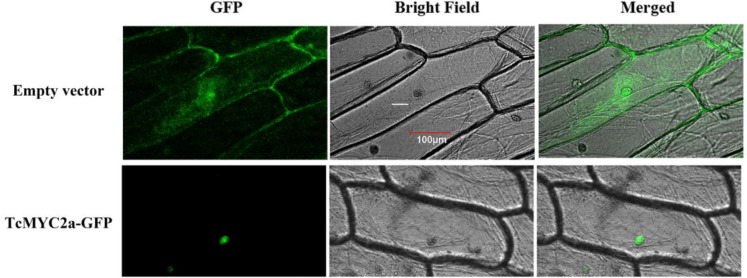
Subcellular localization of TcMYC2a. The fused protein of TcMYC2a and GFP were transformed into onion epidermal cells. GFP fluorescence were observed 2 days after infecting by Laser scanning confocal microscope FV1000 (OLYMPUS, Japan) with 10 × 20 magnification.

Transcription factors always function in nucleus, and MYC2 factors confirm that a nucleus localized signal peptide exists in the JID domain (Uji et al., 2016). Thus, we investigated TcMYC2a localization by expressing TcMYC2a-GFP in onion epidermal cells. Results showed that TcMYC2a actually localized in nucleus (**Figure 4**).

### TcMYC2a Was a Target of *Taxus* JAZ Protein

**FIGURE 5:**
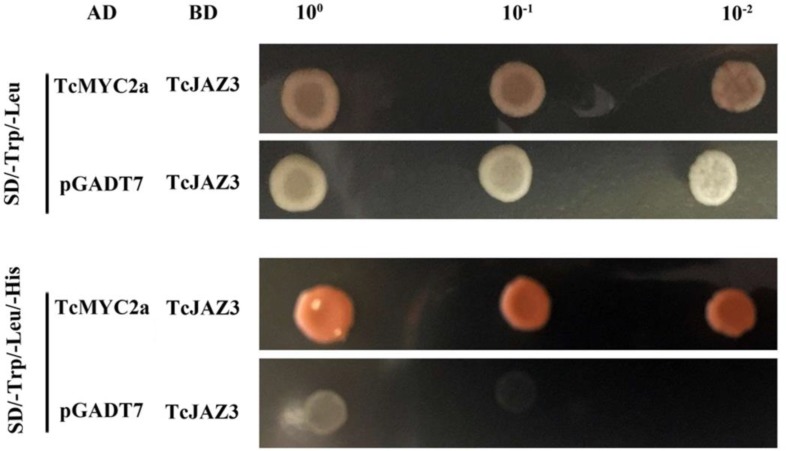
TcMYC2a and TcJAZ3 could physically interacted in Yeast. TcJAZ3 was infused with GAL4 BD in pGBKT7, and TcMYC2a was infused with GAL4 AD in pGADT7. Vacant pGADT7 and TcJAZ3-BD were co-transformed into AH109 as a control.

To verify the roles of TcMYC2a in the JA-response system, TcJAZ3, which was a homolog of AtJAZ3, was used to detect interaction in a yeast two-hybrid system (Zhang et al., 2017). TcMYC2a-AD and TcJAZ3-BD vectors were constructed and then co-transformed into yeast AH109. The vacant AD and TcJAZ3-BD were also co-transformed as a control. Apparently, the positive clones containing both TcMYC2a-AD and TcJAZ3-BD vectors grew on Trp-, Leu-, and His-deficiency media, whereas control clones did not, indicating that TcMYC2a was a target of TcJAZ3 (**Figure 5**).

### TcMYC2a Can Bind With the T/G-, G-Box, and E-Box of *TASY* and *TcERF12/15* Gene Promoters

**FIGURE 6:**
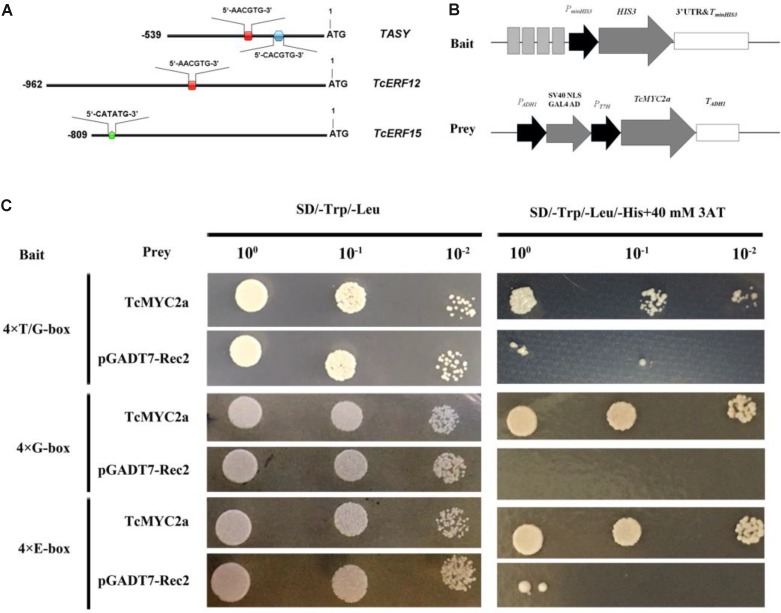
TcMYC2a could bind with T/G-, G- and E-box in yeast. **(A)** The sketch map of promoters of *TASY*, *TcERF12*, and *TcERF15* genes, the red boxes indicated T/G-box (5′-AACGTG-3′), blue hexagon was G-box (5′-CACGTG-3′) and green pentagon was E-box (5′-CATATG-3′). **(B)** Sketch map of the prey vector and bait vectors. The lighter gray boxes indicated T/G-, G-, or E-box, they were synthesized for four times and ligated into pHis2.1; while TcMYC2a was fused with GAL4 AD in pGADT7-Rec2. **(C)** The binding capability tests of TcMYC2a and *cis*-elements in yeast. Related bait vector with each *cis*-element was co-transformed with vacant pGADT7-Rec2 into Y187 as the control.

Previously, a T/G-box (5′-AACGTG-3′; -256 bp to -251 bp) and a G-box (5′-CACGTG-3′; -158 bp to -153 bp) were found in the promoter of *TASY* gene, and deletion analysis indicates that T/G-box is related to JA response (Zhang et al., 2015b). In the present study, 5′-flanking sequences of TcERF12 and TcERF15 were obtained and analyzed with PlantCARE. Results showed a T/G-box (-454 bp to -459 bp) in the 5′ flanking sequence of TcERF12 and E-boxes (CATATG, -736 bp to -741 bp) in the 5′ flanking sequence of TcERF15 (**Figure 6**).

Using quadruple T/G-box, G-box, and E-box as baits, Y187 cells containing bait vector and vacant pGADT7-Rec2 were inhibited when the concentration of 3-AT reached 40 mM in deficiency medium. The yeast Y187 with TcMYC2a-pGADT7-Rec2 vector and bait vector grew well on an SD/-Trp/-Leu/-His+40 mM 3-AT plate (**Figure 6**). These results showed that TcMYC2a can bind with T/G-, G-box, and E-box, indicating that *TASY*, *TcERF12*, and *TcERF15* were the regulating targets of TcMYC2a.

### TcMYC2a Activated the GUS Reporter Gene That Was Under Control of TASY Promoter by Functioning With Either T/G-Box or G-Box

**FIGURE 7:**
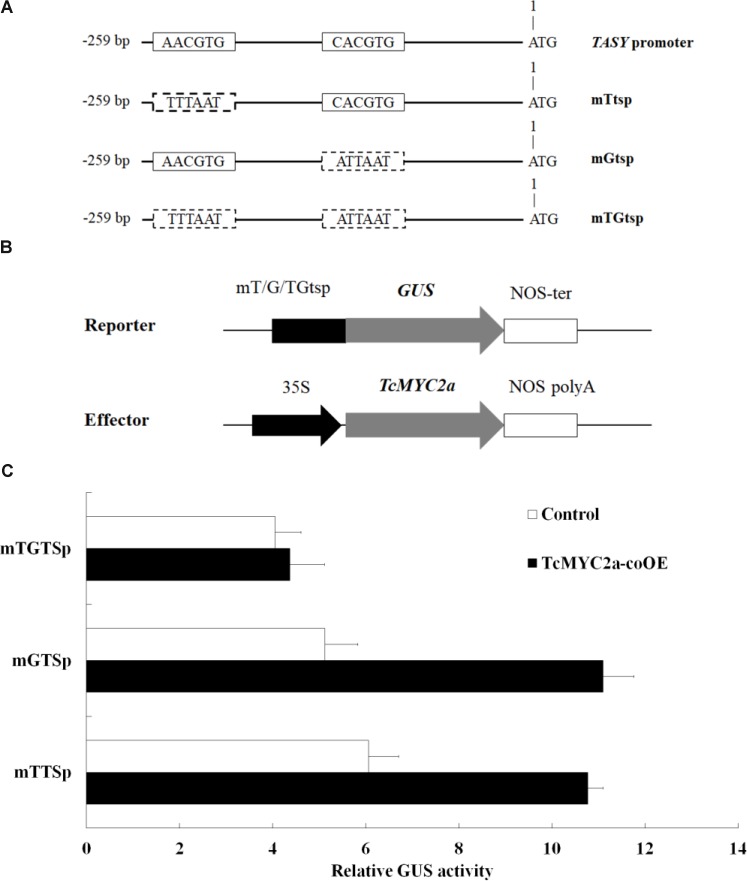
TcMYC2a can transactivate either T/G- or G-box-mediated gene expression in *Taxus* cells. **(A)** Schematic diagram of mutant fragments of *TASY* promoter. *TASY* promoter revealed the original T/G- and G-box, and mTtsp indicated that the T/G-box was mutated by the sequence in the dotted boxes as well as the mGtsp and mTGtsp. **(B)** Schematic diagram of the reporter and effector plasmids used in transient assays. Each of mTtsp, mGtsp, and mTGtsp was fused to the *GUS* gene, respectively. Effector plasmids were under the control of the CaMV 35S promoter. **(C)** Relative GUS activities in TcMYC2a and related reporters co-transformed *Taxus* cells.

Yeast one-hybrid experiment showed that TcMYC2a can bind with T/G- and G-box of *TASY* promoter *in vitro*, indicating that *TASY* gene was a direct target of TcMYC2a. Thus, to confirm the ability of TcMYC2a to bind with *TASY* promoter *in vivo* and analyze its ability in regulating transcription, T/G- and G-box of *TASY* promoter (-259 bp ∼ 0 bp) were mutated and inserted into pBI121 by substituting 35S promoter, respectively, named as mTtsp and mGtsp (**Figure 7**). Moreover, the vector with the fragment containing both mutated T/G- and G-box were used as control and named as mTGtsp. Each of the three vectors were co-transformed with TcMYC2a-p1303 into *Taxus* cells, and GUS activity was detected. Results showed that GUS activity increased 1.78- and 2.18-fold when TcMYC2a was co-expressed with mTtsp and mGtsp, respectively, indicating that TcMYC2a can activate downstream genes by functioning with either T/G-box or G-box (**Figure 7**).

### TcMYC2a Induced Taxol Biosynthesis

**FIGURE 8:**
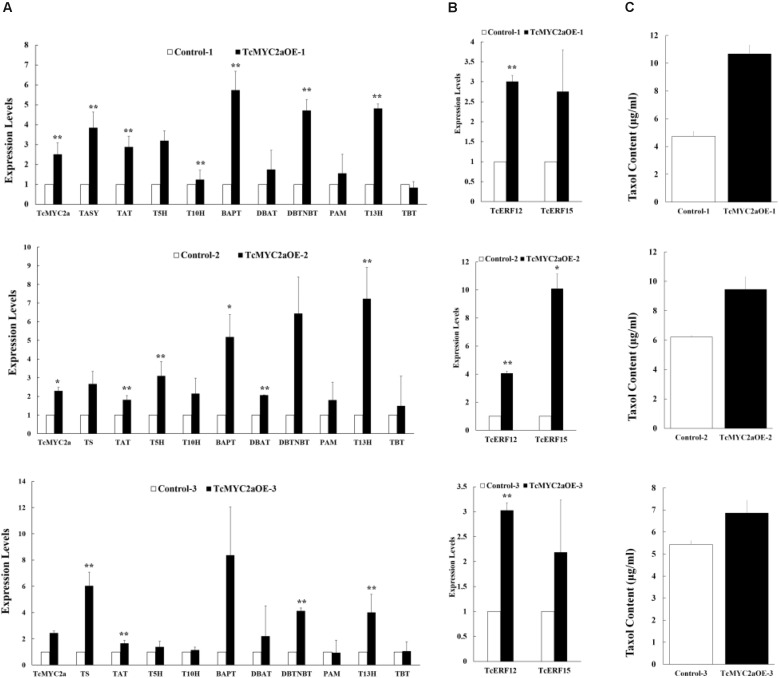
Taxol-biosynthesis related genes were induced in TcMYC2a overexpression cells. The expression of **(A)** taxol biosynthesis genes, **(B)** regulator genes and **(C)** taxol contents (μg/ml) were quantified in TcMYC2a overexpression cell lines by qRT-PCR. Actin was used as reference gene, each experiment was conducted three repeats, and totally three biological repeats were used, the statistics method of *p*-value was Student’s *T*-test. Stars indicated the significance, ^∗^ means that 0.01 < *p*-value < 0.05, ^∗∗^ means that *p*-value < 0.01. TASY (taxadiene synthase), T5H (taxadiene 5-alpha hydroxylase), TAT (taxadienol acetyltransferase), T10H (5-alpha-taxadienol-10-beta-hydroxylase), T13H (13-alpha-hydroxylase gene), TBT (taxane 2-alpha-*O-*benzoyltransferase), DBTNBT (3′-N-debenzoyltaxol *N*-benzoyltransferase), BAPT (phenylpropanoyltransferase), DBAT (10-deacetylbaccatin III-10-O-acetyltransferase) and PAM (phenylalanine aminomutase).

To identify TcMYC2a function in taxol-biosynthesis genes, TcMYC2a was overexpressed in *Taxus* cells. The expression levels of taxol-biosynthesis genes were examined by qRT-PCR. Results showed that *TASY*, *TAT*, *BAPT*, *DBTNBT*, and *T13H* significantly increased in all three TcMYC2a-ovexpression cell lines. Meanwhile, the other genes *T5H*, *T10H*, *DBAT*, and *PAM* were likely to be invariable in TcMYC2a-ovexpression cell lines (**Figure 8A**). Thus, TcMYC2a function was limited compared with JA treatment, which increased nearly all taxol-biosynthesis genes, indicating that TcMYC2a was not the only way for JA signal transduction.

Additionally, we used HPLC-MS to evaluate the content of taxol in TcMYC2a-overexpression cells (**Figure 8C**). The content of taxol was found to be higher in TcMYC2a-overexpression cells than in control samples (Dong et al., 2015).

### TcMYC2a Was an Activator of *TcERF15* in Cells

**FIGURE 9:**
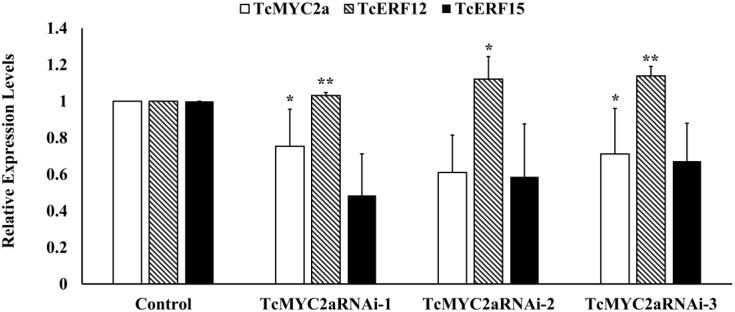
TcERF15 reduced in TcMYC2a-RNAi cells. The data were Statisticsed by Student *T*-test. The stars “*” indicated *p*-value < 0.05, “**” indicated *p*-value < 0.01.

TcERF12 and TcERF15 are reportedly regulators of *TASY* gene and are downstream modules in JA signal response (Zhang et al., 2015b). To clarify the relationship between TcMYC2a and TcERFs in the JA signaling pathway, the expression levels of TcERFs genes were verified in TcMYC2aOE cell lines. qRT-PCR results showed that both TcERF12 and TcERF15 were induced in overexpression cell lines (**Figure 8B**). When TcMYC2a was interfered with by RNAi (RNA interference), only TcERF15 showed a reduction, whereas TcERF12 expressed high stability in transgenic *Taxus* cells (**Figure 9**). All these results indicated that TcERF15 was the dominant target when TcMYC2a transduced JA signals to regulate taxol biosynthesis.

## Discussion

Jasmonate acid signaling pathway is proved to be one of the most important regulating systems in land plants; it is involved in growth, development, (a)biotic defense, and secondary metabolism (Roberts, 2001). The response mechanism to JA signal has been clarified in *Arabidopsis*, *Oryza*, *Nicotiana*, *Solanum*, and other angiosperms, but reports focusing on gymnosperm are few (van der Fits and Memelink, 2000; De Boer et al., 2011; Shoji and Hashimoto, 2011; Zhang et al., 2011, 2012; Zhou et al., 2016). *Taxus* spp. is an ancient gymnosperm conifer that provides us a precious anticancer drug, taxol. Moreover, the JA signaling pathway is one of the most efficient methods regulating taxol biosynthesis (Patil et al., 2012; Onrubia et al., 2013; Zhang et al., 2015b). Therefore, the mechanism by which taxol biosynthesis regulate JA signals can promote our understanding on the significance of the JA signaling pathway in both gymnosperm and angiosperm.

MYC2, which is a bHLH transcription factor, is the key element for transferring JA signals to downstream genes. MYC2 plays important roles in the crosstalk of the JA signaling pathway with other hormone signals (Hong et al., 2012). Recently, MYC2 factors have been discovered as crucial regulators of the JA-mediated activation of secondary-metabolite biosynthesis in medicinal plants, such as NtMYC2a/b, CrMYC2, and AaMYC2. The present study showed that TcMYC2a, first identified in *T. chinensis*, shared high similarity with these known MYC2 factors. Three conserved domains/motif were highly conserved in both TcMYC2a and other MYC2 factors: bHLH-zip-, bHLH-MYC_N-, and BIF/ACT-like-domain. Thus, TcMYC2a was a typical MYC2 factor with similar functions (Pauwels and Goossens, 2011).

The bHLH-zip domain, the common domain of almost all bHLH factors, consists of three conserved motifs, i.e., the basic-region motif responsible for DNA binding (Carretero-Paulet et al., 2010), the Helix–loop–helix motif, and the zip motif involved in homo-/hetero-dimerization (Fernandez-Calvo et al., 2011; Zhou et al., 2016; Lian et al., 2017). Apart from these conserved motifs, TcMYC2a contained the 14-aa co-adjacent peptide, which was conserved only in MYC2 (IIIe) and IIId bHLHs, and a putative phosphorylation site (SELS in TcMYC2a that was homologous with SDHS residues in AtMYC2) was found only in MYC2 factors.

The bHLH-MYC_N-domain was also called MYB interactive region (Pattanaik et al., 2008) and JAZ-interactive domain, which was essential for binding with JAZ and MYB proteins (Fernandez-Calvo et al., 2011; Li et al., 2017). A transcriptional activation domain reportedly required for binding with MED25 in *Arabidopsis* is present in all MYC2 factors (Chen et al., 2012; Zhai et al., 2013). Conserved bHLH-MYC_N-domain factors show similar protein–protein interactions between TcMYC2a and other MYC2, indicating that TcMYC2a has similar functions. The BIF/ACT-like domain is necessary for homo-/hetero-dimerization and function as a switch that permits distinct configurations of a regulatory complex to be tethered to different promoters (Zhang et al., 2003; Kong et al., 2012).

Overall, results of molecular structural analysis of both gymnosperm and angiosperm plants showed that MYC2 transcription factors evolved extremely conservatively, indicating that MYC2 played crucial functions in the plant kingdom. As a homolog of OsMYC2, CrMYC2, AaMYC2a, and NtMYC2a/b, the potential of TcMYC2a in secondary-metabolite biosynthesis in *T. chinensis* was high.

In the JA signaling pathway, JAZ and MYC2 formed the most important heterodimer to control a series of downstream bioactivities by protein–protein interaction, such as AtMYC2 and AtJAZs, NtMYC2b and NtJAZ1/2/3, and AaMYC2 and AaJAZs (Pauwels and Goossens, 2011; Shoji and Hashimoto, 2011; Shen et al., 2016). Previously, we have identified JAZ proteins from several transcriptome datasets of *T. chinensis*. TcJAZs and PsJAZs are found to have several evolutionary differences from AtJAZs and OsJAZs. Among nine TcJAZ proteins, TcJAZ3 was most closely related to AtJAZs (Zhang et al., 2017). The present results showed that TcMYC2a can bind with TcJAZ3 in yeast, indicating that TcMYC2a was a target of JAZ proteins and that the JA signaling pathway in *Taxus* spp. was similar to that in angiosperm plants.

Our previous work has identified the JA-responsive element of *tasy* gene promoter and confirmed that the GCC-box (TGCCGCCT; from -143 to -136 bp), T/G-box (AACGTG; from -256 to -251 bp), and G-box (CACGTG; from -153 to -158 bp) were the effective response regions (Zhang et al., 2015b). AACGTG (T/G-box) and CACGTG (G-box) are classic MYC2-binding sequences (Qi et al., 2015; Du et al., 2017). In the present work, we found that TcMYC2a can bind with both T/G- and G-box in yeast, indicating TcMYC2a can regulate *tasy* gene expression. Results of GUS activity analysis showed that TcMYC2a can positively regulate downstream gene expression by binding with both T/G- and G-box. Subsequently, *TcMYC2a* overexpression in *T. chinensis* cells increased the expression of most taxol biosynthesis genes, especially acyltransferases. HPLC-MS analysis also showed that taxol contents increased in overexpression cells. All these results indicated that TcMYC2a was a positive regulator of *tasy* gene and even most taxol-biosynthesis genes.

However, TcMYC2a was not able to induce all taxol-biosynthesis genes, in contrast to JA stimuli. According to previous studies on taxol induction, JA application can generally promote the expression of taxol-biosynthesis genes in 12 h; for example, *TASY*, *PAM*, *T2H*, *T13H*, and *T7H* can increase several dozen times; *DBAT*, *DBTNBT*, and *BAPT* also increase (Patil et al., 2012; Onrubia et al., 2013). However, the present results showed that only several genes encoding acyltransferases and a few hydroxylation genes were up-regulated in TcMYC2a-overexpression cells, indicating that the JA signaling pathway had more than one way of TcMYC2a dependence (Hu et al., 2013).

*TcMYC2a* overexpression can also increase the expression of *TcERF12* and *TcERF15*, which encode a negative and a positive regulator of *tasy* gene, respectively (Zhang et al., 2015b). RNAi analysis revealed that TcERF12 was not influenced by the down-regulation of TcMYC2a, indicating the possibility of other ways for *TcERF12* to respond to JA stimuli. After 5′-flanking sequence analysis, TcERF12 gene was found to contain many JA-responsive *cis*-elements, such as a W-box (TTGACC; -505 to -500 bp), a T/G-box (CACGTT/AACGTG; -454 to -459 bp), and MYB-binding sites (CAGTTA; -63 to -58 bp). Considering the presence of these elements, TcMYC2a was capable of activating TcERF12 directly or indirectly in accordance with the results of TcMYC2a-overexpression analysis. In tobacco, MYC2 factors simultaneously activate negative and positive regulators, and down-regulation is observed for ERF189 as a negative regulator and other positive clade 2 ERFs in NtMYC2-RNAi plants (Shoji and Hashimoto, 2011). To prevent hypersensitive JA response, many researchers have reported that negative and positive factors are up-regulated simultaneously because they are essential to the balance of various bioactivities in plants (Nakata et al., 2013). Meanwhile, distinct preferences in DNA binding with the GCC-box are possessed by ERF repressors and activators, indicating that they regulate different targets in natural plants (Fujimoto et al., 2000; Zhang et al., 2015b).

In wild-type *Taxus* cells, *TcERF15* seemed to be the key regulating target of TcMYC2a when transducing JA signals to taxol-biosynthesis genes. All these results indicated that TcMYC2a can transduce JA signals and regulate taxol biosynthesis by activating *TcERF15* expression. In other plants, MYC2 factors regulate the biosynthesis of secondary metabolites in several ways. NtMYC2a/b can up-regulate biosynthesis genes and regulators, whereas CrMYC2 up-regulates biosynthesis genes by up-regulating regulators (De Boer et al., 2011; Zhang et al., 2011). Interestingly, physical interactions and cooperation between OsMYC2 and other factors enhance their transactivation activity (Ogawa et al., 2017a,b). Our results indicated that TcMYC2a had a similar functional mode with NtMYC2a/b. Additionally, interactors and cooperative regulators of TcMYC2a and known MYC2s should be further identified, so the dual functions of MYC2 factors should be clarified.

**FIGURE 10:**
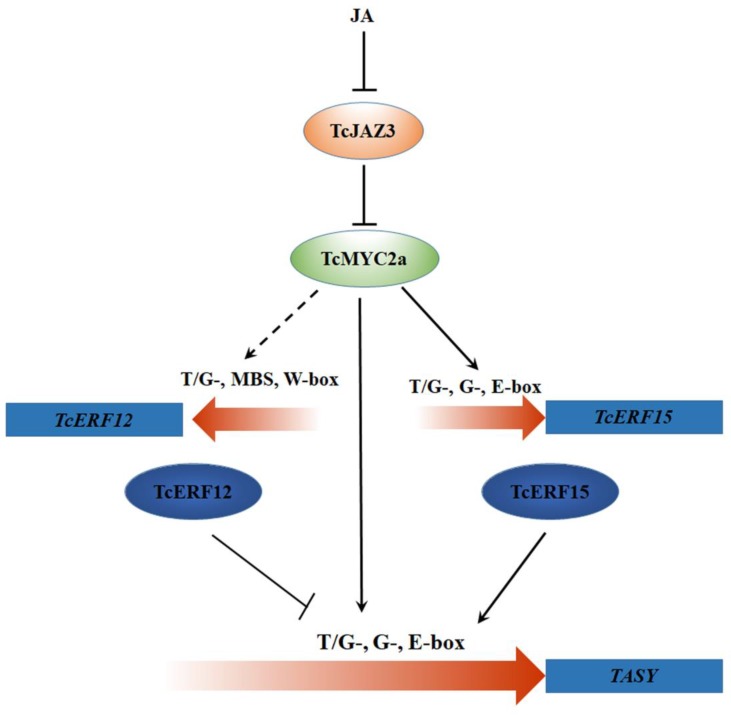
TcMYC2a-mediated regulation pathways of taxol biosynthase genes by jasmonate (JA). The dotted line indicated the potential regulation. TcJAZ3, *Taxus chinensis* jasmonate zim-domain transcription factors; TcMYC2a, *Taxus chinensis* MYC2 transcription factor; TcERF12 and TcERF15, *Taxus chinensis* ethylene responsive factors; T/G-, G-box and E-box, *cis*-element in gene promoter region; TASY, taxadiene synthase.

All these results indicated two regulating pathways of TcMYC2a (**Figure 10**): (i) TcMYC2 can directly bind with T/G-, G-, and E-box present in promoters and then activate the expression of taxol-biosynthesis genes; and (ii) TcMYC2a can activate the expression of transcriptional factors to regulate downstream genes. These results were highly accordance to those observed in tobacco, *C. roseus*, and tomato (Shoji and Hashimoto, 2011; Zhang et al., 2011; Du et al., 2017).

Furthermore, TcMYC2a was proved to be the crucial element for transducing JA signals to downstream responsive genes and then positively regulate taxol biosynthesis either directly or by regulating ERF regulators. Our results indicated that *Taxus* had a similar JA signaling pathway to angiosperm plants, supposing that the JA signaling pathway was highly conservatively evolved land plants.

## Data Availability

All sequences obtained in this paper has submitted into GenBank, and their accession numbers are MG494378 (TcMYC2a), MG494379 (TcERF12p), and MG494380 (TcERF15p).

## Author Contributions

MZ, CF, and LY conceived and designed the research. MZ, XJ, YC, SZ, MW, and WL conducted the experiments. MZ analyzed the data and wrote the manuscript. All authors read and approved the manuscript.

## Conflict of Interest Statement

The authors declare that the research was conducted in the absence of any commercial or financial relationships that could be construed as a potential conflict of interest.

## Footnotes

1 http://pfam.sanger.ac.uk

2 http://hmmer.org/

3 http://www.ncbi.nlm.nih.gov/BLAST/

4 https://npsa-prabi.ibcp.fr/cgi-bin/npsa_automat.pl?page=/NPSA/npsa_clustalw.html

5 http://espript.ibcp.fr/ESPript/cgi-bin/ESPript.cgi

6 ftp://ftp.ncbi.nlm.nih.gov/blast/executables/blast+/LATEST/

7 http://bioinformatics.psb.ugent.be/webtools/plantcare/html/

8 http://www.dna.affrc.go.jp/PLACE/signalscan.html

## Supplementary Material

TABLE S1 Primers and synthesized fragments used in this paper. All primers used in this paper. The primers names started with dl were used for qRT-PCR. The other primers were used for vector construction and gene amplification, and the restriction sites indicated the restriction enzymes used in vector constructing. mGtsp was synthesized by Sangon Biotech (Shanghai) Co., Ltd. The restriction sites in TGbF, TGbR, GbF, GbR, EbF, and EbR were designed since the cohesive end would expose after annealing.

## Abbreviations

BAPT: phenylpropanoyltransferase
bHLH: basic helix loop helix
DBAT: 10-deacetylbaccatin III-10-*O*-acetyltransferase
DBTNBT: 3′-*N*-debenzoyltaxol *N*-benzoyltransferase
JA: jasomonate acid
PAM: phenylalanine aminomutase
T10H: 5-alpha-taxadienol-10-beta-hydroxylase
T13H: 13-alpha-hydroxylase gene
T5H: taxadiene 5-alpha hydroxylase
TASY: taxadiene synthase
TAT: taxadienol acetyltransferase
TBT: taxane 2-alpha-*O*-benzoyltransferase
zip: leucine zipper
